# The effect of medical grade compression garments on the repeated‐bout effect in non‐resistance‐trained men

**DOI:** 10.1113/EP091399

**Published:** 2023-09-28

**Authors:** Freddy Brown, Matt Hill, Derek Renshaw, Charles Pedlar, Jessica Hill, Ken van Someren, Glyn Howatson, Jason Tallis

**Affiliations:** ^1^ School of Life Sciences Coventry University Coventry UK; ^2^ Research Centre for Physical Activity, Sport and Exercise Science Coventry University Coventry UK; ^3^ Centre for Health and Life Sciences Coventry University Coventry UK; ^4^ Faculty of Sport, Health and Applied Science St Mary's University Twickenham UK; ^5^ Institute of Sport, Exercise and Health, Division of Surgery and Interventional Science University College London London UK; ^6^ Sports Lab Northwest Atlantic Technological University Donegal Ireland; ^7^ Faculty of Health and Life of Sciences Northumbria University Newcastle Upon Tyne UK; ^8^ Water Research Group Northwest University Potchefstroom South Africa

**Keywords:** adaptation, compression, muscle damage, performance, recovery, strength

## Abstract

Whilst compression garments (CG) may enhance recovery from exercise‐induced muscle damage (EIMD), many recovery strategies can attenuate adaptative responses. Therefore, the effects of CG on recovery from EIMD, and the rapid protective adaptations known as the repeated bout effect (RBE) were investigated. Thirty‐four non‐resistance‐trained males (18–45 years) randomly received class II medical‐grade CG or placebo for 72 h following eccentrically‐focused lower‐body exercise, in a double‐blind, randomised controlled trial. Indices of EIMD were assessed at baseline, 0, 24, 48 and 72 h post‐exercise, before exercise and testing were repeated after 14 days. Results were analysed using a three‐way (time × condition × bout) linear mixed‐effects model. Exercise impaired isometric and isokinetic strength, with soreness and thigh circumference elevated for 72 h (*P* < 0.001). Compression did not enhance recovery (*P* > 0.05), despite small to moderate effect sizes (ES, reported alongside 90% confidence intervals) for isokinetic strength (ES from 0.2 [−0.41, 0.82] to 0.65 [0.03, 1.28]). All variables recovered faster after the repeated bout (*P* < 0.005). However, RBE for peak isokinetic force was impaired in CG at 60° s^−1^ (group × bout interaction: χ^2^ = 4.24, *P* = 0.0395; ES = −0.56 [−1.18, 0.07]) and completely absent at 120° s^−1^ (χ^2^ = 16.2, *P* < 0.001, ES = −0.96 [−1.61, −0.32]) and 180° s^−1^ (χ^2^ = 10.4, *P* = 0.001, ES = −0.72 [−1.35, −0.09]). Compression blunted RBE at higher isokinetic velocities without improving recovery in non‐resistance‐trained males, potentially contraindicating their use following unaccustomed exercise in this population.

## Abstract (highlights)

- **What is the central question of this study?**
  What are the effects of compression garments on recovery from unaccustomed damaging exercise and subsequent protective adaptations?
- **What is the main finding and its importance?**
  Compression did not influence recovery, but was associated with blunted protective adaptations for isokinetic performance, which were completely absent at high velocities. Based on these findings, the use of compression garments for recovery would not be recommended following unaccustomed exercise, particularly if the maintenance of high‐velocity performance following exercise‐induced muscle damage is desirable.

## 1 INTRODUCTION

The concept of recovery describes the reestablishment of post‐exercise performance, with important implications for recreational exercisers and athletes alike (Peake, 2019; Skorski et al., 2019). Sufficient recovery allows athletes to achieve and maintain an optimal training stimulus (Peake, 2019; Skorski et al., 2019), whilst perceptual symptoms of under‐recovery may reduce adherence in recreational exercisers (Flack et al., 2017). Importantly, however, some anti‐inflammatory and/or antioxidant recovery strategies have been shown to ameliorate the physiological stressors required for muscular adaptation (Figueiredo et al., 2016; Peake, 2019). The use of particular recovery strategies may therefore represent a compromise between optimising short‐term recovery and maximising the response to training.

The time course of post‐exercise recovery depends upon the specific physiological demands of an exercise bout (Skorski et al., 2019), with exercise‐induced muscle damage (EIMD) contributing to prolonged declines in muscular performance (Nosaka et al., 2006). Describing the disruption of muscle fibres and associated reductions in contractile force, EIMD may be associated with strength deficits of up to 30% in untrained participants (Clarkson & Hubal, 2002; Nosaka et al., 2006) and is most commonly caused by unaccustomed, eccentric (muscle‐lengthening) exercise, such as plyometrics, resistance exercise and running (Brown et al., 2017; Goto & Morishima, 2014; Hill et al., 2017; Thomas et al., 1995). The progression of EIMD is mediated by post‐exercise inflammation (Deyhle et al., 2016; Hyldahl et al., 2017), with symptoms such as soreness and impaired mobility persisting for up to 10 days (Clarkson & Hubal, 2002). One seemingly effective recovery strategy for EIMD is the use of compression garments (CG), which has been associated with ameliorated strength losses, swelling and cellular disruption in both trained and untrained participants (Brown et al., 2017; Marques‐Jimenez et al., 2016). However, the effects of CG on adaptation have received little attention (Baum et al., 2020; Edgar et al., 2022). Robust data on the effects of compression on muscular adaptation are required to guide the use of CG for recovery.

Muscle‐damaging exercise is unique in that as little as a single exposure may elicit adaptations which confer protection from subsequent bouts (Hyldahl et al., 2017). This ‘repeated bout effect’ (RBE) may be observed after as little as 3 days and is most pronounced following unaccustomed exercise (Chen & Nosaka, 2006; Hyldahl et al., 2017). Although the mechanisms responsible for RBE are unclear, post‐exercise inflammation is thought to at least partially mediate the protective adaptations and modified inflammatory responses to EIMD (Deyhle et al., 2016; Hyldahl et al., 2017). Importantly, researchers have suggested that the benefits of CG may be related to an ameliorative effect on local inflammation (Hill et al., 2017; Peake, 2019), with anti‐inflammatory effects reported in both sporting (Valle et al., 2013) and clinical settings (Beidler et al., 2009). As compression may influence mediators of the repeated bout effect, the effects of CG on RBE require further investigation.

Whilst the use of CG for recovery from EIMD is supported by academic consensus, the literature is still beset by the varied quality of supporting evidence (Brown et al., 2017; Marques‐Jimenez et al., 2016). For example, most studies have failed to implement a placebo or sham condition (reviewed by Brown et al., 2017), or where used, to report on the effectiveness of blinding (Brown et al., 2017; de Glanville & Hamlin, 2012; Hill et al., 2017). The few double‐blind studies that exist have compared CG to non‐compressive garments – a ‘sham treatment’ of questionable efficacy (Baum et al., 2020; de Glanville & Hamlin, 2012). Although previous meta‐analyses have reported large benefits to strength recovery when CG are worn following EIMD (Brown et al., 2017; Marques‐Jimenez et al., 2016), the placebo effect can explain benefits of this magnitude (Clark et al., 2000). Such issues prevent researchers from making informed, quantitative judgements on the benefits of CG, compared with the potential risks to adaptation. Accordingly, the aims of this investigation were to examine the effects of CG on recovery from EIMD using a double‐blind approach, and to determine the effects of compression on RBE in non‐resistance‐trained participants. Given the ambiguity of existing evidence and uncertainties over the mechanisms involved, results were compared to a two‐tailed null hypothesis – that no differences in recovery or RBE would be observed.

## 2 METHODS

### 2.1 Ethical approval

Institutional ethics approval was obtained from Coventry University Ethics Board (Ref. P93660) before written informed consent was provided by all participants. The study conformed to the standards set by the *Declaration of Helsinki*, except for registration in a database.

### 2.2 Design

The effects of CG on muscular recovery and RBE following EIMD were assessed using a double‐blind, three‐way (condition × time × bout) design. Analysis was performed unblinded to allow assessment of participant adherence. Performance measures and indices of EIMD were taken at baseline, and then at 0, 24, 48 and 72 h (±2 h) after an initial bout of exercise (B1) to assess recovery in participants wearing CG or taking placebo (PLA). A repeated bout (B2) was completed after 14 days without any recovery intervention, and recovery monitored at the same time points. To account for the highly individual (Clarkson & Hubal, 2002; Hyldahl et al., 2017), and potentially sex‐specific (Fernandez‐Gonzalo et al., 2014), nature of EIMD responses, only male participants were recruited, and pair‐matched following familiarisation according to body mass and the number of weekly exercise bouts of various types that were undertaken (self‐report). Exercise was categorised into running, non‐load‐bearing exercise (swimming and cycling) and multi‐directional exercise (e.g. team sports) to account for the protective effects of prior damage, which is greater following load‐bearing activity (Thomas et al., 1995). Participants were also matched by body mass to control for the effects of total work and eccentric forces on EIMD responses to the load‐bearing exercise challenge employed (depth‐jumps) (Paschalis et al., 2005). Participants were then randomly assigned to either group, using open‐access statistical software (‘optmatch’ package; R Foundation for Statistical Computing, Vienna, Austria).

### 2.3 Participants

Selecting an α‐value of 0.05 with 80% statistical power, a sample size calculation was carried out with the G*Power software package (version 3.1.9.7, Heinrich Heine University, Düsseldorf, Germany), based upon an expected Cohen's *d* effect size of 1 from previous studies assessing the effects of CG on recovery from EIMD on knee‐extension performance (Goto & Morishima, 2014; Hill et al., 2017). While a matched‐pair design required a minimum sample of *n* = 10 (2 × *n* = 5), a conservative estimate of 34 (2 × *n* = 17) was chosen, based on an independent group design. Accordingly, 34 males (18−45 years) who met national physical activity guidelines (Care, 2019) were recruited (Table 1). Those who had completed lower‐body resistance exercise within 6 months were excluded to maximise the likelihood of observing RBE (Deyhle et al., 2016; Hyldahl et al., 2017), as were participants whose habitual exercise had been disrupted by injury for at least 1 week of the preceding 28 days. Participants were asked to refrain from taking non‐steroidal anti‐inflammatory drugs or supplements (including antioxidants and whey protein) throughout testing, from 48 h before the start of the study.

**TABLE 1:** Participant characteristics.

	CG	PLA
Age (years)	27.3 ± 6.7	25.1 ± 8.5
Body mass (kg)	77 ± 11.3	78.1 ± 10.4d
Stature (m)	1.78 ± 0.1	1.77 ± 0.1
Σ8 skinfolds (mm)	89 ± 44.8	112 ± 55.5
MVIC (N)	639 ± 122	635 ± 141
MTG (cm)	55.3 ± 5.9	56.2 ± 4.6
Calf (cm)	37.5 ± 2	37.9 ± 2.3
CG pressure (mmHg)		
Thigh	16 ± 3	
Calf	23 ± 6	
MB1	21 ± 5	
Ankle	16 ± 2	

Ankle, interface pressure taken at point of narrowest circumference of the ankle; Calf, point of widest circumference at the medial calf; MB1, manufacturer's B1 point; Thigh, mid‐thigh skinfold site. Abbreviations: CG, compression garments; MVIC, maximal isometric voluntary contraction; MTG, mid‐thigh girth; PLA, placebo.

### 2.4 Procedures

Familiarisation was conducted over two sessions, 1–2 weeks before the trial. Body mass was measured (Seca 875 Class III scales, Seca Medical Measuring Systems, Birmingham, UK), before anthropometry was assessed in accordance with the recommendations of the International Society for the Advancement of Kinanthropometry by a level 1 practitioner. Skinfolds (Σ8), mid‐thigh girth (MTG) and calf circumference were measured in a standing position, with the latter used to guide the selection of appropriately sized British class II (moderate) compression stockings, according to manufacturer guidelines (Duomed soft thigh length compression stockings, Medi UK Ltd, Hereford, UK). Subsequently, CG were measured for applied pressures in all participants (Picopress, Microlab, Padua, Italy) at the mid‐thigh, medial calf, ankle (Bjork & Ehmann, 2019) and ‘manufacturer's B1 point’ (MB1) (Uhl et al., 2013). To ensure physiologically relevant pressures, smaller CG were provided if stockings failed to apply 14 mmHg at the thigh – a proposed threshold in the literature (Hill et al., 2015). The entire battery of performance tests was then completed in the same order as subsequent experimental trials (Brown et al., 2023).

**FIGURE 1:**
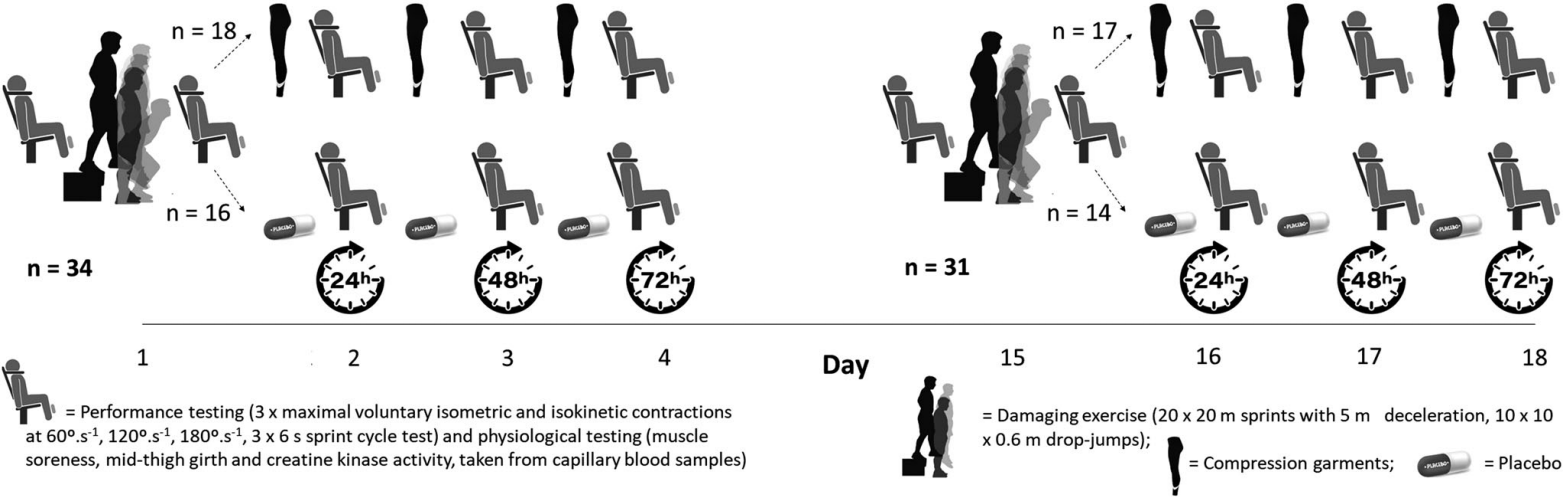
Study design and procedures.

After a standardised warm‐up (3 min cycling at 100 W; 10 repetitions of body‐weight squats, lunges on either leg and countermovement jumps), peak force was assessed from three (5 s) maximal isometric voluntary contractions (MIVC) of the knee extensors at 85° flexion (KinCom, Chattanooga, TN, USA – 100 Hz), followed by three consecutive isokinetic contractions at each of 60, 120 and 180° s^−1^ (Figure 1). Participants sat reclined at 15°, were secured with straps, and were prevented from gripping the chair during contractions. Finally, peak power output in the 6 s cycle sprint test (PPT) was assessed in a standing position from a stationary start (Brown et al., 2023). Participants were positioned so that the knee was just flexed at the bottom of the stroke when seated (handlebars adjusted for comfort), with settings recorded and repeated for each visit. Resistance was determined from manufacturer settings (Wattbike Pro, Wattbike Ltd, Nottingham, UK). Verbal encouragement was given for all tests. A minimum of three repetitions per test was completed during each familiarisation session, or until performance plateaued as defined by the final two efforts resulting in coefficients of variation (CV) ≤ 5%. Between‐day reliability values (CV) were 6% for MVIC and 9.4%, 3.5% and 7.5% for peak isokinetic force at 60, 120 and 180° s^−1^, respectively. Within‐session CVs were 6%, 8.9%, 5.5% and 5.4%. Peak cycle sprint power was described by CVs of 4.8% and 3.9% for between‐day and within‐session values, respectively.

At baseline, soreness, MTG and capillary blood samples from the finger were taken as previously described (Brown et al., 2022), with changes in MTG taken as a measure of post‐exercise swelling (Brown et al., 2017; Goto & Morishima, 2014; Marques‐Jimenez et al., 2016). Performance was assessed as above, taking peak values from the best of three attempts separated by 90 s recovery. Serum was assessed for creatine kinase (CK) activity using spectrophotometric assay with a between‐run precision of <1%, as determined in our laboratory (CK‐NAC, RX Daytona, Randox, County Antrim, Northern Ireland). A repeated sprint protocol (Duffield et al., 2010) (20 × 20 m sprints with a 5 m deceleration) followed by 10 sets of 10 depth‐jumps (DJs) was then completed (Hill et al., 2017) to induce EIMD (Figure 1). Participants completed sprints every 60 s, with sprint times (TC PhotoGate, Brower timing Systems, UT, USA) relayed to participants to encourage maximal effort. Sets of 10 DJs were completed every 2.5 min from a 0.6 m box onto two force plates sampling at 1000 Hz (AMTI BP900900, Watertown, MA, USA) to measure vertical ground reaction forces. Jump height was determined from take‐off velocity for each jump using forward integration (McMahon et al., 2021), first calculating impact velocity from the 0.6 m descent. Peak and average forces during breaking and propulsion, as well as the work performed for each jump (equivalent to the sum of average breaking and propulsive forces multiplied by countermovement depth), were calculated to characterise the exercise challenge (Table 2), alongside starting and peak heart rates for each sprint and set of DJs (S810iTM, Polar, Kempele, Finland). Participants were told they would have to repeat any sprint where they continued beyond the marked 5 m deceleration zone, and any DJ for which the knee angle at the bottom of the jump failed to reach 90° flexion.

**TABLE 2:** Exercise responses from a combined repeated‐sprint (20 × 20 m) and depth‐jump (10 × 10) muscle‐damage protocol.

	CG		PLA	
	B1	B2	B1	B2
Repeated sprints				
20 m sprint time (s)	3.66 ± 0.47	3.56 ± 0.27	3.53 ± 0.2	3.5 ± 0.24
HR_med_ (beats min^−1^)	146 ± 15	144 ± 20	144 ± 21	140 ± 19
HR_pk_ (beats min^−1^)	155 ± 15	148 ± 15	154 ± 18	151 ± 17
Depth‐jumps				
Breaking *F*_pk_ (N)	4463 ± 1005	4263 ± 710	4743 ± 895	4606 ± 857
Breaking *F*_ave_ (N)	1424 ± 123	1421 ± 75	1436 ± 67	1447 ± 77
Propulsive *F*_pk_ (N)	1648 ± 198	1682 ± 211	1600 ± 207	1631 ± 207
Propulsive *F*_ave_ (N)	1085 ± 99	1086 ± 58	1075 ± 72	1046 ± 62
Jump height TOV (cm)	13 ± 2	13 ± 1	12 ± 1	13 ± 1
Total work (J)	176904 ± 284	184384 ± 173	163703 ± 167	176416 ± 184
Total work (J kg^−1^ per jump)	23 ± 4	24 ± 2	22 ± 2	22 ± 2
HR_med_ (beats min^−1^)	156 ± 14	150 ± 16	153 ± 18	152 ± 18
HR_pk_ (beats min^−1^)	170 ± 12	164 ± 16	166 ± 16	166 ± 18

CG, compression garments; PLA, placebo; B1, Bout 1; B2, repeated bout; HR_med_, median heart‐rate; HR_pk_, peak heart‐rate; *F*_pk_, peak force; *F*_ave_, average force; TOV, as calculated from take‐off velocity.

Following B1, participants received either CG or placebo tablets providing <0.1 g carbohydrate (6 mm hard lactose/sucrose tablets, HSC, Holt, UK) and instructed to either consume a tablet or don CG in private before leaving the building. Garments were worn for 72 h post‐exercise, with participants instructed to remove CG only to wash, and before arriving for subsequent testing. Participants assigned to PLA were given three tablets to consume (daily) immediately after testing, having been told by a researcher that they contained magnesium to aid recovery. Participants were randomised into groups A and B by the lead researcher, and informed of their specific interventions by a third party. To aid placebo blinding, participants were told there was an additional control group (group C) for comparison. This group did not exist, and participants were informed of this deception at the conclusion of data collection. Participants were requested to record their dietary intake from the day before the trial until 72 h post‐exercise, and to replicate this for the repeated bout. The effectiveness of blinding was assessed at the conclusion of the trial by asking participants to rate their intervention for perceived efficacy from 0 to 10, accepting half‐marks (Karanicolas et al., 2010). Finally, baseline performance tests, eccentrically focused exercise and assessments of recovery were repeated after 14 d to investigate RBE.

### 2.5 Statistical analysis

An open‐access statistical software package was used for all statistical analyses (R Foundation for Statistical Computing, Vienna, Austria), which were carried out on raw data (although changes in MTG were graphically presented as normalised values for clarity). Despite initial efforts at pair‐matching, follow‐up questioning on adherence revealed that one participant mistakenly took the wrong allocation, resulting in *n* = 18 and *n* = 16 for CG and PLA, respectively. Accordingly, a between‐group design was employed, using a linear mixed effects model with random intercepts for each participant to account for individual variation in EIMD (Gandotra et al., 2021). The consideration of random effects in a statistical model also allows for participants to be included up until the point of withdrawal to enhance statistical power and reduce sample bias (Nich & Carroll, 2002). Visually comparing fitted and observed values, and calculating root mean squared error (RMSE) from residuals, revealed this model better matched observed data for every variable compared with analysis of variance (ANOVA). The assumption of normality was verified by running QQ plots on residual values, with the mixed effects model independent of the assumption of sphericity. *Post hoc* testing was carried out with the ‘emmeans’ package, adjusting for multiple comparisons (Gandotra et al., 2021). Ordinal soreness data were assessed with a non‐parametric alternative to the split‐plot ANOVA (Feys, 2016). All values were reported as means ± SD. Additionally, effect sizes (ES; Cohen's *d*) were calculated as between‐group differences in changes from baseline, and reported alongside 90% confidence intervals as (ES [LCL, UCL]), where LCL and UCL represent lower and upper 90% confidence limits. Effect sizes were defined as trivial/small, moderate or large using thresholds of ≥0.2, ≥0.5 and ≥0.8, respectively (Batterham & Hopkins, 2006). *Post hoc* power assessments were carried out for all performance tests using G*Power.

## 3 RESULTS

Due to COVID‐19 pandemic restrictions (*n* = 1) and injuries during the second exercise bout (*n* = 2), data on the repeated bout were missing for three participants (CG, *n* = 1; PLA, *n* = 2). Baseline participant characteristics and garment pressures are detailed below, alongside exercise responses (Tables 1 and 2), which did not differ between bouts or groups at either time point (*P* > 0.05). Perceived efficacy did not differ (*P* = 0.558) between CG (5 ± 2) and PLA (4.5 ± 2), suggesting blinding was effective. Stockings were worn for 20.9 ± 3.7 h day^−1^, while participants in PLA each consumed all three tablets.

**FIGURE 2:**
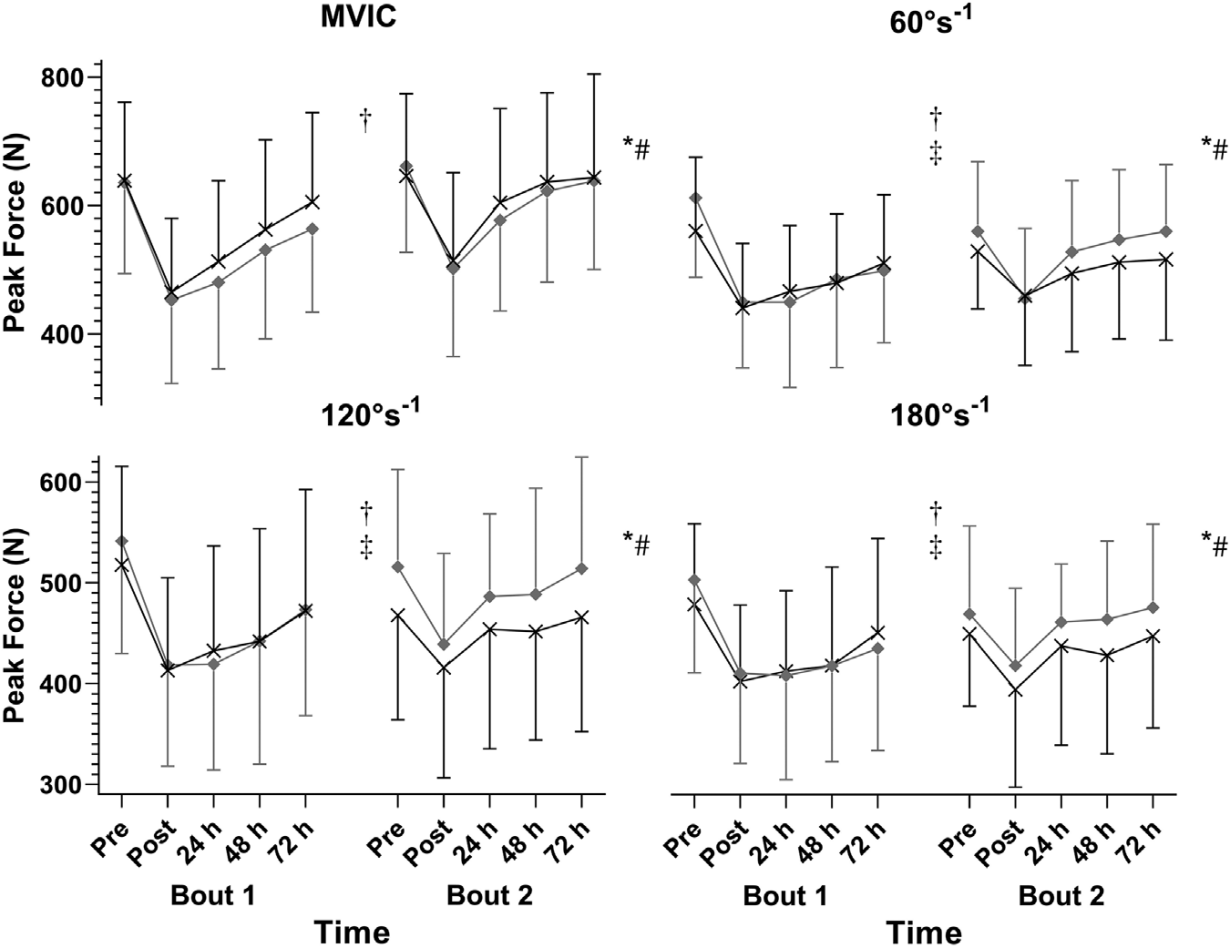
Peak maximal voluntary contraction force for isometric (MVIC) and isokinetic contractions at 60, 120 and 180° s^−1^, over an initial (left) and repeated bout (right) of eccentrically focused exercise. Black line and crosses, compression garments; grey line and diamonds, placebo. *Significant effect of time (*P* < 0.001 for each variable); †significant difference between bouts (*P* < 0.001 for each variable); #significant time × bout interaction (*P* < 0.001 for all isokinetic speeds, *P* = 0.004 for MVIC); ‡significant bout × group interaction (*P* = 0.0395, *P* < 0.001, *P* = 0.001 for 60° s^−1^, 120° s^−1^ and 180° s^−1^, respectively; MVIC: *P* = 0.128).

Muscle damage and RBE were apparent from post‐exercise declines in all isometric and isokinetic strength measurements, which were attenuated between bouts, and recovered more rapidly in B2 (Figure 2). For MVIC, significant effects were observed for time (χ^2^ = 268, *P* < 0.001), bout (χ^2^ = 84.3, *P* < 0.001) and their interaction (χ^2^ = 15.6, *P* = 0.004), without differences between groups (*P* > 0.05 – Figure 2). Performance was impaired for 72 h following B1 (post – 48 h: *P* < 0.001; 72 h, *P* = 0.006) but had recovered by 48 h after B2 (*P* = 1 at each time point – Supplementary Table), with peak force higher at all post‐exercise time points (*P* < 0.001–0.005).

Peak isokinetic forces at 60, 120 and 180° s^−1^ (Figure 2) were subject to significant effects (*P* < 0.001) of time (χ^2^ = 182, 184 and 92.7, respectively) and bout (χ^2^ = 22.6, 15.0, 14.0), as well as time × bout interactions (χ^2^ = 35.1, 36.7, 25.0). Demonstrating RBE, performance following B1 was impaired for 72 h at each velocity (*P* < 0.001), whereas strength recovered by 24 h following B2 (*P =* 0.1165, *P* = 0.3568, *P* = 1). Accordingly, peak force was greater in B2 at each velocity from 24–72 h post‐exercise (*P* < 0.001–0.0186). Of note, baseline performance was also lower (*P* = 0.0071, *P* = 0.0026 and *P* = 0.0343 for 60, 120 and 180° s^−1^, respectively). Neither group nor time × group effects were apparent, with the moderate improvements observed in CG following B1 at 60 and 180° s^−1^ failing to reach significance (*P* > 0.05, ES from 0.51 [−0.11, 1.13] to 0.65 [0.03, 1.28] – Table 3). Observed RBE for isokinetic strength was lower in CG than PLA for each velocity, as shown by significant bout × group interactions (*P* < 0.001–0.0395; (Figure 2, Table 3, Supplementary Table). At 60° s^−1^, although performance deterioration was attenuated following B2 in both CG (*P* = 0.0336) and PLA (*P* < 0.001), RBE was significantly smaller in CG (χ^2^ = 4.24, *P* = 0.0395; ES = −0.56 [−1.18, 0.07]; (Figure 2, Table 3, Supplementary Table). Conversely, the bout × group interactions observed at 120° s^−1^ (χ^2^ = 16.2, *P* < 0.001) and 180° s^−1^ (χ^2^ = 10.4, *P* = 0.001) revealed that RBE was absent in CG. Whilst *post hoc* testing demonstrated greater peak forces in PLA following B2 at both 120 and 180° s^−1^ (*P* < 0.001 for both), no increase was observed in CG (*P* = 0.875, *P* = 0.5507; Figure 2, Table 3, Supplementary Table).

**TABLE 3:** Effect sizes and 90% confidence limits for between group differences.

Time	Post	24 h	48 h	72 h	RBE
MVIC	0.09 [−0.53, 0.7]	0.34 [−0.28, 0.96]	0.27 [−0.35, 0.88]	0.33 [−0.28, 0.95]	−0.38 [−1, 0.23]
IKD 60° s^−1^	0.4 [−0.22, 1.01]	0.65 [0.02, 1.27]	0.46 [−0.16, 1.08]	0.65 [0.03, 1.28]	−0.56 [−1.18, 0.07]
IKD 120° s^−1^	0.24 [−0.38, 0.86]	0.48 [−0.14, 1.11]	0.25 [−0.36, 0.87]	0.2 [−0.41, 0.82]	‐0.96 [−1.61, −0.32]
IKD 180° s^−1^	0.32 [−0.29, 0.94]	0.38 [−0.24, 1]	0.3 [−0.32, 0.92]	0.51 [−0.11, 1.13]	‐0.72 [−1.35, −0.09]
PPT	0.32 [−0.3, 0.94]	0.25 [−0.36, 0.87]	0.41 [−0.21, 1.03]	0.15 [−0.47, 0.76]	0.18 [−0.43, 0.8]
MTG	0.4 [−0.22, 1.01]	0.65 [0.02, 1.27]	0.46 [−0.16, 1.08]	0.65 [0.03, 1.28]	0.16 [−0.45, 0.78]
Soreness	−0.08 [−0.69, 0.54]	−0.39 [−1.01, 0.23]	−0.02 [−0.63, 0.59]	0.09 [−0.53, 0.7]	0.09 [−0.53, 0.7]
CK activity	−0.47 [−1.09, 0.15]	0.09 [−0.52, 0.7]	−0.22 [−0.84, 0.39]	−0.35 [−0.97, 0.27]	0.33 [−0.29, 0.95]

Effect sizes (Cohen's *d*) reported as ES [LCL, UCL]), where LCL and UCL represent the lower and upper 90% confidence limits. Effect size thresholds were as follows: ≤ 0.2 (trivial), 0.2–0.49 (small), 0.5−0.79 (moderate), >0.8 (large). CK, creatine kinase; IKD, maximal voluntary isokinetic contraction; MTG, mid‐thigh girth; MVIC, maximal voluntary isometric contraction; Post, post‐exercise; PPT, peak power output in the 6 s cycle sprint test; RBE, repeated bout effect (defined as the difference between the means of the repeated and the initial bout).

A decline in PPT was observed over time (χ^2^ = 153, *P* < 0.001), with peak power recovering by 48 h (*P* = 1). Cycle power improved over B2 compared to B1 (*P* < 0.001). *Post hoc* power analyses at individual time points following B1 yielded β values of 0.14−0.58 for force dynamometry, and 0.11−0.32 for peak cycling power. Power from observed differences for RBE between groups ranged from 0.29 to 0.86 for force dynamometry and was 0.13 for PPT.

**FIGURE 3:**
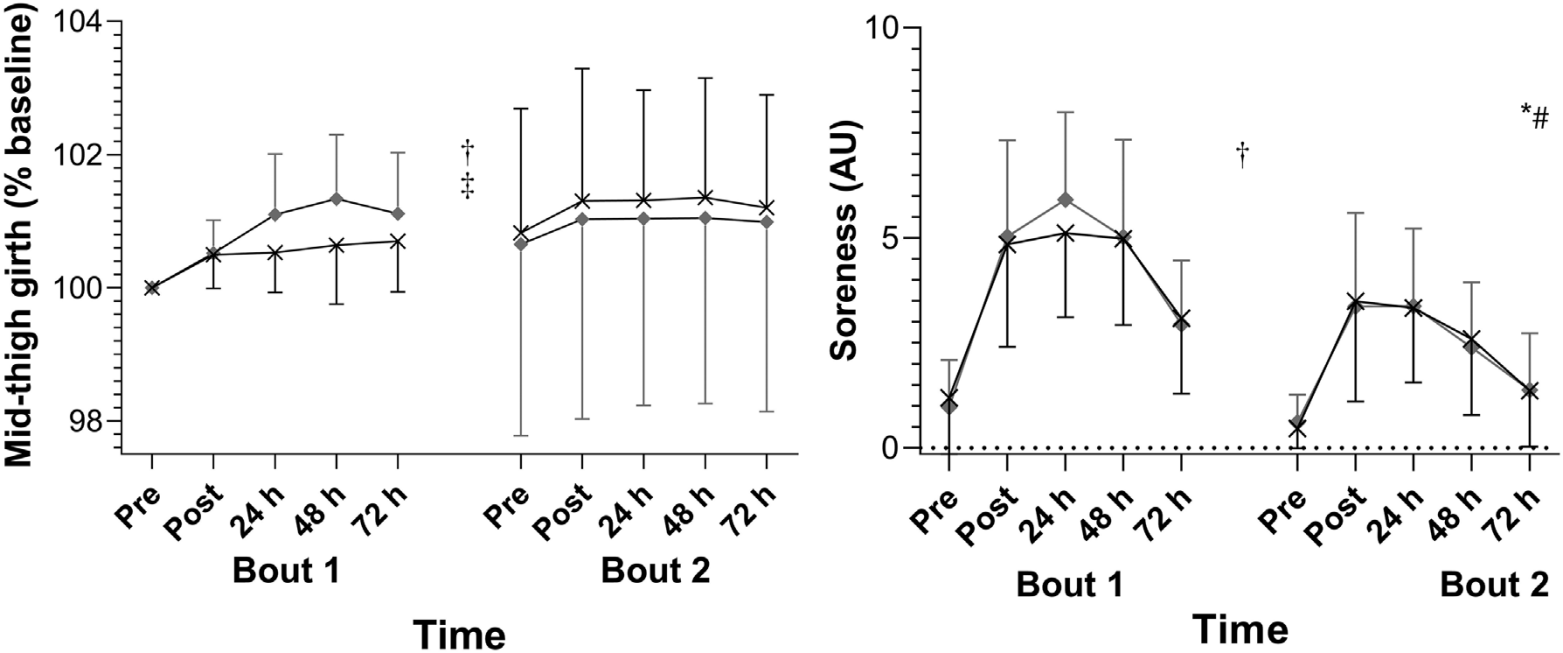
Mid‐thigh girth and soreness over an initial (left) and repeated bout (right) of eccentrically focused exercise. Black line and crosses, compression garments; grey line and diamonds, placebo. *Significant effect of time (*P* < 0.001); †significant difference between bouts (mid‐thigh girth: *P* = 0.004; soreness: *P* < 0.001); ‡significant bout × group interaction (*P* = 0.01); #significant time × bout interaction (*P* < 0.001).

Changes in MTG were observed over time (χ^2^ = 14.2, *P* < 0.001) and between bouts (χ^2^ = 8.25, *P* = 0.004), with mean values greater than baseline from 24 h (*P =* 0.049, *P =* 0.0114 and *P =* 0.0382 for 24 h, 48 and 72 h, respectively – Figure 3, Supplementary Table). None of group × time (χ^2^ = 1.15, *P* < 0.886), three‐way (χ^2^ = 1.26, *P* = 0.868), nor bout × time interactions were significant (χ^2^ = 0.585, *P* = 0.630). However, a bout × group effect was observed (χ^2^ = 6.66, *P* = 0.01), with *post hoc* testing demonstrating that swelling in CG was greater following B2, when compression was not worn (*P* < 0.001). Conversely, swelling did not differ between bouts in PLA (*P* = 0.984). Soreness (Figure 3) changed over time (χ^2^ = 2.87, *P* < 0.001) and between bouts (χ^2^ = 62.8, *P* < 0.001), and demonstrated a significant time × bout interaction (χ^2^ = 6.3, *P* < 0.001). No significant differences or interactions between groups were apparent (*P* > 0.05). *Post hoc* testing revealed that while soreness remained elevated from baseline at all times during B1 (*P* < 0.001), values returned to baseline by 72 h in B2 (*P* = 0.151).

In addition to participant attrition, CK analysis was limited further by insufficient blood samples from one participant, with a further two participants giving anomalous pre‐exercise readings. Technical error was ruled out by analysing dilutions of the original sample, which all indicated similar CK activity (within 1%−15%), while elevated baseline readings did not reflect impaired muscular performance compared to the previous/subsequent bout. These anomalous readings resulted in between‐bout differences at least 3 times greater than the group SD of the differences (exceeding the 99% confidence interval), so were removed from the analysis. Creatine kinase activity in the resulting 28 participants changed over time χ^2^ = 16.7, *P* = 0.002), and between bouts (χ^2^ = 12.1, *P* < 0.001), with no other differences or interactions observed (*P* > 0.05). Hypothesis testing was not affected by the inclusion or exclusion of the anomalous results (main effects for set and bout; χ^2^ = 11.9, *P* = 0.0179 and χ^2^ = 13.3, *P* < 0.001, respectively). *Post hoc* testing revealed that average values at 24 h were greater than those recorded at both pre‐exercise (*P* = 0.0124) and post‐exercise (*P* = 0.0444) time points.

## 4 DISCUSSION

The present study investigated the effects of CG on recovery and adaptation following EIMD. Whilst the effects of CG on recovery were not significant, RBE for isokinetic performance was impaired for each velocity. Although these findings were specific to isokinetic strength, these data provide novel and robust evidence that CG may undermine aspects of RBE in non‐resistance‐trained males.

The current findings do not support previous studies which suggest that CG enhance recovery from EIMD (Brown et al., 2017; Hill et al., 2017; Marques‐Jimenez et al., 2016; Peake, 2019). These observations may be related to several discrepancies between the current study and previous trials – including differences in interface pressures, populations and exercise protocols (Brown et al., 2017; Marques‐Jimenez et al., 2016). However, as the placebo effect can enhance performance by up to 6% (ES = 1.2) (Clark et al., 2000), it is also possible that the effective blinding strategy employed was at least partly responsible (Table 3). Effective blinding has also been postulated to explain non‐significant findings on cold water immersion which contradict those from earlier studies (Wilson et al., 2018). Furthermore, the current study was conducted double‐blind to eliminate the possibility of providing subconscious cues to participants, which can further inflate treatment effects (Karanicolas et al., 2010). These data highlight the need to implement and evaluate a double‐blind approach to elucidate the true effects of CG on recovery.

The non‐significant effects of CG for recovery in the current study are somewhat surprising, as observed interface pressures were similar to previously proposed pressure optima. For example, benefits have been commonly observed from garments applying around 14−20 mmHg at the thigh, above or below which pressures CG may be ineffective (Hill et al., 2017; Miyamoto & Kawakami, 2014). A 15% improvement in recovery of countermovement jump performance was previously reported in recreationally active participants (Hill et al., 2017) when class II CG, providing almost identical interface pressures to those we report, were worn for 72 h following 100 DJs (14.8 ± 2.2 vs. 16 ± 3 mmHg at the thigh and 24.3 ± 3.7 vs. 23 ± 6 at the calf). However, pressure optima have yet to be conclusively established (Brown et al., 2017; Hill et al., 2017, 2015). Furthermore, even directly measured interface pressures may vary with small changes in sensor placement and non‐uniformities in limb profile (Bjork & Ehmann, 2019), complicating comparisons between trials. Error also arises from the different sensors used, with the Kikuhime device used by Hill et al. (2017) known to overestimate applied pressures by 10−15% compared to the Picropress (Partsch & Mosti, 2010). Discrepancies may also be related to differences in the recovery of jumping versus isometric performance (Byrne & Eston, 2002), or the mixed sex population studied by Hill et al., considering potential sex‐specific responses to EIMD (Clarkson & Hubal, 2002; Fernandez‐Gonzalo et al., 2014). Further research is required to establish pressure optima for recovery, with a pressing need for researchers in the field to report directly measured pressures using standardised procedures.

The findings we present are the first to report deleterious effects from CG on muscular adaptation, contradicting previous findings which suggest variable, but likely positive effects on strength, power and endurance outcomes (Baum et al., 2020; Edgar et al., 2022). However, the adaptations underpinning RBE are likely distinct from those elicited by either hypertrophy or endurance training (Hyldahl et al., 2017). Additionally, the compression stimulus provided in the current trial likely differed from previous studies. The military recruits in Edgar's study wore compression leggings for 4–6 h day^−1^ only, with reported interface pressures (15.0 ± 4.3 and 11.8 ± 3.1 mmHg at the thigh, before and after the training intervention, respectively) measured with the Kikuhime. No placebo was given to the control group. Conversely, Baum et al. (2020) studied the effects of sports compression leggings worn *during* training (pressures not reported), presumably to investigate the cumulative effects of enhanced training performance. The data we present suggest the negative effects of medical‐grade CG on RBE are greater than any potential benefits for recovery following unaccustomed exercise in non‐resistance‐trained males.

The current findings are the first to demonstrate negative effects from compression on adaptation – specifically on RBE for isokinetic strength. Furthermore, these effects appear to be velocity‐specific, with RBE reduced at 60° s^−1^, but completely absent in CG at 120 and 180° s^−1^. That these observations were made following the use of an effective placebo intervention, double‐blind design, and reported alongside a reduction in swelling following B1 strengthens the case for a physiologically mediated effect of CG, with the combined α value from all three isokinetic interactions equating to *P <* 3.95 × 10^−8^. These findings are also concordant with previous observations that protective adaptations to EIMD are associated with a greater relative recruitment of oxidative motor‐units and subsequent preservation of high velocity performance (Hinks et al., 2021; Hortobagyi et al., 1996; Hyldahl et al., 2017). In the current study, however, these adaptations were absent in CG. Although it is important to note that no other indices of EIMD were blunted (e.g. isometric strength or CK), these findings have important implications for the use of compression throughout unaccustomed training. The utility of strength training for improving high‐velocity performance is well established (Cronin et al., 2007; García‐Valverde et al., 2022), with greater isokinetic strength commonly associated with improved power performance in active and athletic populations (Janicijevic et al., 2020; Moreira et al., 2021).

Whilst the mechanisms involved were not explicitly investigated, nor inflammation directly measured, several physiological observations in the current study are concordant with previous findings suggesting that CG reduce tissue damage by moderating inflammation (Beidler et al., 2009; Valle et al., 2013). Interestingly, impaired RBE in CG was observed following an attenuated swelling response after B1 (Figures 2, 3). As swelling is known to propagate the inflammatory response by facilitating leukocyte adhesion (Lawrence & Springer, 1991), and RBE is thought to be mediated by inflammation (Deyhle et al., 2016; Figueiredo et al., 2016), it is possible these two observations are related. Although CG have not been shown to reliably reduce circulating inflammatory markers when worn for recovery (Duffield et al., 2010; Hill et al., 2017; Peake, 2019), previous findings suggest CG can moderate leukocyte infiltration; which was reduced in professional footballers 48 h after CG were worn *during* downhill‐running (Valle et al., 2013). Research is required to establish the biochemical effects of compression, the relevance to recovery and specific adaptations.

### 4.1 Limitations

The current study is subject to a number of important limitations, most notably the small sample size. The double‐blind (initially pair‐matched) design made it inappropriate to replace participants who withdrew, while the initial sample size was calculated from single blind studies, which may overestimate treatment effects (Karanicolas et al., 2010). Interestingly, isokinetic performance at baseline was lower in B2 at all three velocities, despite the fact that RBE manifested as greater mean values over B2 due to more rapid recovery. This may suggest that either recovery following B1 was incomplete, or that having undergone a previous bout of damaging exercise, participants were dissuaded from providing maximal efforts. Indeed, an apparent trend for lower baseline performance in a repeated bout has been reported previously (Falvo et al., 2010). However, it is important to note that this lower baseline for B2 did not differ between groups (no three‐way interaction), and was not observed for MIVC (a criterion measure for assessing EIMD). As such, this observation does not affect our conclusion – that RBE was attenuated in CG.

Another limitation of the current study is the loss of CK data, which may also have masked meaningful differences. The non‐significant 31–62% reductions in CG are perhaps noteworthy considering the variable nature of this measure (Clarkson & Hubal, 2002; Hill et al., 2017; Paschalis et al., 2005). Individual EIMD responses were also highly variable, with pre–post declines in isometric strength varying from 5.3% to 59.6% in this sample. Similarly, the lack of effects from CG on soreness are also likely due in part to the highly subjective nature of this measure (Fitzgerald et al., 1991). Finally, as RBE is population and exercise specific (Hyldahl et al., 2017), it would be unwise to extrapolate these findings to other scenarios. Research on females and studies investigating the effects of compression pressure on strength and endurance adaptations are required.

### 4.2 Conclusion

Compression garments did not enhance muscular recovery in non‐resistance‐trained males, although RBE for isokinetic strength was impaired. These findings provide the only evidence to date that the use of CG attenuates muscular adaptation. Based on these findings, the use of CG for recovery would not be recommended following unaccustomed exercise, particularly if the maintenance of high‐velocity performance and resilience to EIMD are desirable.

## AUTHOR CONTRIBUTIONS

Freddy Brown, Jessica Hill, Charles Pedlar, Glyn Howatson and Ken van Someren conceived and designed the research. F.B. conducted experiments, acquiring and analysing all data, with J.T. contributing analytical tools. Freddy Brown, Jason Tallis, Matt Hill, Derek Renshaw, Glyn Howatson and Ken van SomerenKen van Someren all contributed to interpreting the results. Freddy Brown analysed data and wrote the manuscript, with support in drafting and critically revising the article provided by all authors. All authors have read and approved the final version of this manuscript and agree to be accountable for all aspects of the work in ensuring that questions related to the accuracy or integrity of any part of the work are appropriately investigated and resolved. All persons designated as authors qualify for authorship, and all those who qualify for authorship are listed.

## CONFLICT OF INTEREST

The authors have no competing interests to declare.

## FUNDING INFORMATION

The authors did not receive support or funding from any organisation for the submitted work.

## Supporting information

Supplementary Table

## Data Availability

The data that support the findings of this study are available from the corresponding author upon reasonable request.
