# Supplementary material for: The effect of medical grade compression garments on the repeated‐bout effect in non‐resistance‐trained men
Source: Exp Physiol. 2023 Sep 28;108(12):1490–9. doi: 10.1113/EP091399 (PMC10988506; doi:10.1113/EP091399)
Supplement: Supplementary file 1 — Supplementary Table [file EPH-108-1490-s001.docx]

| **Supplementary Table. Raw and relative values for indices of muscle damage over time** | | | | | | | | | | | | | | | | | | | | |  |
| --- | --- | --- | --- | --- | --- | --- | --- | --- | --- | --- | --- | --- | --- | --- | --- | --- | --- | --- | --- | --- | --- |
|  |  | **Bout 1** | | | | | **Bout 2** |  |  |  |  | **T** | | **B** | | **T x B** | | **G x B** | |  |  |
| **Measure** | **Time** | **Pre** | **Post** | **24 h** | **48 h** | **72 h** | **Pre** | **Post** | **24 h** | **48 h** | **72 h** | ***p*** | | ***p*** | | ***p*** | | ***p*** | |  |  |
| **MVIC (N)** | CG | 639 ± 122 | 465 ± 115 | 513 ± 126 | 563 ± 140 | 606 ± 139 | 646 ± 128 | 514 ± 137 | 604 ± 146 | 637 ± 139 | 644 ± 161 |  | |  | |  | |  | |  |  |
|  | PLA | 635 ± 141 | 453 ± 130 | 480 ± 135 | 531 ± 138 | 563 ± 129 | 661 ± 135 | 502 ± 137 | 577 ± 141 | 622 ± 142 | 638 ± 138 | *** | | *** | | ** | |  | |  |  |
| **IKD 60⁰.s^-1^ (N)** | CG | 560 ± 115 | 440 ± 100 | 466 ± 102 | 479 ± 107 | 510 ± 106 | 528 ± 89 | 459 ± 108 | 494 ± 121 | 511 ± 119 | 516 ± 125 |  | |  | |  | |  | |  |  |
|  | PLA | 611 ± 123 | 450 ± 102 | 450 ± 132 | 485 ± 137 | 498 ± 111 | 559 ± 108 | 455 ± 109 | 528 ± 111 | 546 ± 109 | 560 ± 104 | *** | | *** | | *** | | * | |  |  |
| **IKD 120⁰.s^-1^ (N)** | CG | 518 ± 98 | 413 ± 92 | 433 ± 104 | 442 ± 112 | 472 ± 121 | 468 ± 104 | 416 ± 109 | 454 ± 119 | 451 ± 107 | 466 ± 113 |  | |  | |  | |  | |  |  |
|  | PLA | 542 ± 112 | 419 ± 100 | 419 ± 105 | 442 ± 122 | 473 ± 105 | 516 ± 97 | 439 ± 90 | 486 ± 82 | 488 ± 106 | 514 ± 111 | *** | | *** | | *** | | *** | |  |  |
| **IKD 180⁰.s^-1^ (N)** | CG | 480 ± 81 | 403 ± 76 | 413 ± 80 | 419 ± 98 | 451 ± 94 | 450 ± 72 | 395 ± 97 | 438 ± 99 | 429 ± 98 | 448 ± 91 |  | |  | |  | |  | |  |  |
|  | PLA | 504 ± 92 | 411 ± 90 | 409 ± 104 | 419 ± 96 | 436 ± 102 | 470 ± 88 | 419 ± 77 | 462 ± 58 | 465 ± 78 | 477 ± 83 | *** | | *** | | *** | | *** | |  |  |
| **PPT (W)** | CG | 1016 ± 205 | 918 ± 211 | 964 ± 221 | 1010 ± 223 | 1027 ± 217 | 1058 ± 232 | 990 ± 220 | 1061 ± 240 | 1079 ± 223 | 1101 ± 226 |  | |  | |  | |  | |  |  |
|  | PLA | 1017 ± 146 | 884 ± 184 | 941 ± 163 | 975 ± 141 | 1014 ± 144 | 1038 ± 154 | 947 ± 155 | 1021 ± 171 | 1046 ± 154 | 1069 ± 149 | *** | | *** | |  | |  | |  |  |
| **MTG (cm)** | CG | 54.5 ± 4.5 | 54.8 ± 4.5 | 54.8 ± 4.5 | 54.9 ± 4.5 | 54.9 ± 4.7 | 54.7 ± 4.8 | 55 ± 4.9 | 54.9 ± 4.8 | 55 ± 4.8 | 54.9 ± 4.9 |  | |  | |  | |  | |  |  |
|  | PLA | 55.5 ± 4.4 | 55.8 ± 4.4 | 56.1 ± 4.6 | 56.2 ± 4.6 | 56.1 ± 4.7 | 55.9 ± 4 | 56.1 ± 3.9 | 56.1 ± 4 | 56.1 ± 4 | 56.1 ± 4 | *** | | ** | |  | | ** | |  |  |
| **SOR (AU)** | CG | 1.2 ± 1.3 | 4.8 ± 2.4 | 5.1 ± 2 | 5 ± 2.1 | 3.1 ± 1.8 | 0.5 ± 0.5 | 3.5 ± 2.4 | 3.3 ± 1.8 | 2.6 ± 1.8 | 1.4 ± 1.3 |  | |  | |  | |  | |  |  |
|  | PLA | 1 ± 1.1 | 5 ± 2.3 | 5.9 ± 2.1 | 5 ± 2.3 | 2.9 ± 1.5 | 0.6 ± 0.7 | 3.4 ± 2.2 | 3.4 ± 1.9 | 2.4 ± 1.6 | 1.4 ± 1.4 | *** | | *** | | *** | |  | |  |  |
| **[CK] (IU)** | CG | 264 ± 189 | 347 ± 226 | 1351 ± 1372 | 833 ± 839 | 672 ± 356 | 260 ± 160 | 305 ± 149 | 505 ± 229 | 380 ± 212 | 374 ± 447 |  | |  | |  | |  | |  |  |
|  | PLA | 360 ± 239 | 468 ± 228 | 1249 ± 1159 | 1205 ± 1427 | 1755 ± 4229 | 321 ± 159 | 398 ± 168 | 657 ± 283 | 451 ± 195 | 355 ± 124 | ** | | *** | |  | |  | |  |  |
| **Values as percentage of baseline** | | | | | | | | | | | | |  | |  | |  | |  | | |
|  |  | **Bout 1** |  |  |  |  | **Bout 2** |  |  |  |  |  | |  | |  | |  | |  |  |
| **Measure** | **Time** | **Pre** | **Post** | **24 h** | **48 h** | **72 h** | **Pre** | **Post** | **24 h** | **48 h** | **72 h** |  | |  | |  | |  | |  |  |
| **MVIC** | CG | 100 ± 0 | 73 ± 12 | 80 ± 13 | 88 ± 13 | 94 ± 12 | 102 ± 9 | 82 ± 19 | 95 ± 13 | 100 ± 12 | 101 ± 11 |  | |  | |  | |  | |  |  |
|  | PLA | 100 ± 0 | 71 ± 15 | 76 ± 14 | 84 ± 15 | 90 ± 15 | 109 ± 13 | 83 ± 19 | 95 ± 20 | 103 ± 19 | 105 ± 17 |  | |  | |  | |  | |  |  |
| **IKD 60⁰.s^-1^** | CG | 100 ± 0 | 79 ± 14 | 84 ± 13 | 86 ± 12 | 92 ± 10 | 97 ± 14 | 84 ± 14 | 89 ± 13 | 92 ± 11 | 93 ± 11 |  | |  | |  | |  | |  |  |
|  | PLA | 100 ± 0 | 74 ± 11 | 74 ± 17 | 79 ± 16 | 83 ± 16 | 95 ± 15 | 78 ± 17 | 90 ± 19 | 92 ± 13 | 95 ± 13 |  | |  | |  | |  | |  |  |
| **IKD 120⁰.s^-1^** | CG | 100 ± 0 | 80 ± 11 | 83 ± 12 | 85 ± 11 | 91 ± 13 | 91 ± 9 | 81 ± 13 | 88 ± 11 | 87 ± 10 | 90 ± 10 |  | |  | |  | |  | |  |  |
|  | PLA | 100 ± 0 | 77 ± 11 | 78 ± 12 | 81 ± 15 | 88 ± 13 | 98 ± 11 | 84 ± 14 | 93 ± 10 | 93 ± 12 | 97 ± 10 |  | |  | |  | |  | |  |  |
| **IKD 180⁰.s^-1^** | CG | 100 ± 0 | 84 ± 7 | 86 ± 10 | 87 ± 12 | 94 ± 13 | 95 ± 8 | 83 ± 13 | 91 ± 12 | 89 ± 11 | 94 ± 9 |  | |  | |  | |  | |  |  |
|  | PLA | 100 ± 0 | 81 ± 9 | 81 ± 15 | 83 ± 12 | 87 ± 15 | 96 ± 13 | 86 ± 13 | 95 ± 8 | 95 ± 13 | 98 ± 13 |  | |  | |  | |  | |  |  |
| **PPT** | CG | 100 ± 0 | 90 ± 9 | 95 ± 10 | 99 ± 9 | 101 ± 8 | 104 ± 6 | 98 ± 11 | 105 ± 10 | 107 ± 11 | 109 ± 10 |  | |  | |  | |  | |  |  |
|  | PLA | 100 ± 0 | 87 ± 13 | 92 ± 8 | 96 ± 6 | 100 ± 7 | 104 ± 9 | 95 ± 12 | 103 ± 12 | 105 ± 11 | 108 ± 10 |  | |  | |  | |  | |  |  |
| **MTG** | CG | 100 ± 0 | 100.5 ± 0.5 | 100.5 ± 0.6 | 100.6 ± 0.9 | 100.7 ± 0.8 | 100.8 ± 1.9 | 101.3 ± 2 | 101.3 ± 1.7 | 101.4 ± 1.8 | 101.2 ± 1.7 |  | |  | |  | |  | |  |  |
|  | PLA | 100 ± 0 | 100.5 ± 0.5 | 101.1 ± 0.9 | 101.3 ± 1 | 101.1 ± 0.9 | 100.7 ± 2.9 | 101 ± 3 | 101 ± 2.8 | 101 ± 2.8 | 101 ± 2.8 |  | |  | |  | |  | |  |  |
| **[CK]** | CG | 100 ± 0 | 139 ± 25 | 627 ± 741 | 402 ± 484 | 323 ± 228 | 122 ± 69 | 153 ± 114 | 255 ± 197 | 179 ± 105 | 195 ± 282 |  | |  | |  | |  | |  |  |
|  | PLA | 100 ± 0 | 146 ± 40 | 495 ± 682 | 482 ± 646 | 700 ± 1692 | 113 ± 71 | 145 ± 84 | 244 ± 175 | 159 ± 97 | 129 ± 75 |  | |  | |  | |  | |  |  |

Results shown as mean ± SD; * = p ≤ 0.05; ** = p ≤ 0.01; *** = p ≤ 0.001; T = effect of time; B = effect of bout; T x B = Time x bout interaction; G x B = Group x bout interaction; CG = Compression garments; PLA = Placebo; Pre = Pre-exercise; Post = Post-exercise; MVIC = Maximal voluntary isometric contraction; IKD = Maximal voluntary isokinetic contraction; PPT = peak power output in the 6 s cycle sprint test; MTG = Mid-thigh girth; AU = Arbitrary units; [CK] = Creatine kinase activity; IU = International units
